# CarDiac magnEtic Resonance for prophylactic Implantable cardioVerter defibrillAtor ThErapy in Non-Dilated Left Ventricular Cardiomyopathy: a sub-study from the DERIVATE registry

**DOI:** 10.1093/ehjci/jeaf043

**Published:** 2025-02-03

**Authors:** Isabella Leo, Santo Dellegrottaglie, Alessandra Scatteia, Daniele Torella, Raffaele Abete, Giovanni Donato Aquaro, Andrea Baggiano, Andrea Barison, Jan Bogaert, Leonardo Calo’, Giovanni Camastra, Samuela Carigi, Nazario Carrabba, Grazia Casavecchia, Stefano Censi, Gloria Cicala, Carlo N De Cecco, Manuel De Lazzari, Gabriella Di Giovine, Monica Dobrovie, Marta Focardi, Laura Fusini, Nicola Gaibazzi, Annalaura Gismondi, Matteo Gravina, Marco Guglielmo, Chiara Lanzillo, Massimo Lombardi, Valentina Lorenzoni, Jordi Lozano-Torres, Davide Margonato, Chiara Martini, Francesca Marzo, Pier-Giorgio Masci, Ambra Masi, Claudio Moro, Giuseppe Muscogiuri, Saima Mushtaq, Alberto Nese, Alessandro Palumbo, Anna Giulia Pavon, Patrizia Pedrotti, Martina Perazzolo Marra, Silvia Pradella, Cristina Presicci, Mark G Rabbat, Claudia Raineri, Jose’ F Rodriguez-Palomares, Stefano Sbarbati, Uwe Joseph Schoepf, Angelo Squeri, Nicola Sverzellati, Rolf Symons, Emily Tat, Mauro Timpani, Giancarlo Todiere, Adele Valentini, Akos Varga-Szemes, Alessandra Volpe, Andrea Igoren Guaricci, Juerg Schwitter, Gianluca Pontone

**Affiliations:** Advanced Cardiovascular Imaging Unit, Clinica Villa dei Fiori, Acerra (Naples), Italy; Department of Experimental and Clinical Medicine, Magna Graecia University, Catanzaro, Italy; Advanced Cardiovascular Imaging Unit, Clinica Villa dei Fiori, Acerra (Naples), Italy; Advanced Cardiovascular Imaging Unit, Clinica Villa dei Fiori, Acerra (Naples), Italy; Department of Experimental and Clinical Medicine, Magna Graecia University, Catanzaro, Italy; Department of Cardiology, Policlinico di Monza, Monza, Italy; U.O.C. Risonanza Magnetica per Immagini, Fondazione G. Monasterio CNR-Regione Toscana Pisa, Pisa, Italy; Department of Perioperative Cardiology and Cardiovascular Imaging, Centro Cardiologico Monzino IRCCS, Via Carlo Parea, 4 , Milan 20138, Italy; U.O.C. Risonanza Magnetica per Immagini, Fondazione G. Monasterio CNR-Regione Toscana Pisa, Pisa, Italy; Department of Radiology, University Hospital Leuven, Leuven, Belgium; Cardiology Department, Policlinico Casilino, Rome, Italy; Cardiac Department, Vannini Hospital Rome, Rome, Italy; Department of Cardiology, Infermi Hospital, Rimini, Italy; Cardiotoracovascular Department, Careggi Hospital, Florence, Italy; Department of Medical and Surgical Sciences, University of Foggia, Foggia, Italy; MariaCecilia Hospital, GVM Care & Research, Cotignola, RA, Italy; Department of Diagnostic, Parma University Hospital, Via Gramsci, Parma, Italy; Division of Cardiothoracic Imaging, Emory University, Atlanta, GA, USA; Department of Cardiac, Thoracic, Vascular Sciences and Public Health University of Padua Medical School, Padova, Italy; Department of Cardiology, Policlinico di Monza, Monza, Italy; Department of Radiology, University Hospital Leuven, Leuven, Belgium; Division of Cardiology, Department of Medical Biotechnologies, University of Siena, Siena, Italy; Department of Perioperative Cardiology and Cardiovascular Imaging, Centro Cardiologico Monzino IRCCS, Via Carlo Parea, 4 , Milan 20138, Italy; Department of Electronics, Information and Bioengineering, Politecnico di Milano, Italy; Department of Cardiology, Azienda Ospedaliero-Universitaria, Parma, Italy; Division of Cardiology, Department of Medical Biotechnologies, University of Siena, Siena, Italy; Department of Radiology, University of Foggia, Foggia, Italy; Division of Heart and Lungs, Department of Cardiology, Utrecht University, Utrecht, The Netherlands; Department of Cardiology, Haga Teaching Hospital, The Hague, The Netherlands; Cardiology Department, Policlinico Casilino, Rome, Italy; Multimodality Cardiac Imaging Section, IRCCS Policlinico San Donato, San Donato Milanese, Milan, Italy; Institute of Management, Scuola Superiore Sant’Anna, Pisa, Italy; Department of Cardiology, Hospital Universitari Vall d’Hebron, Barcelona, Spain; Vall d’Hebron Institut de Recerca (VHIR), Universitat Autònoma de Barcelona, Bellaterra, Spain; CIBER-CV, Instituto de Salud Carlos III (ISCIII), Madrid, Spain; Department of Cardiology, Policlinico di Monza, Monza, Italy; Department of Diagnostic, Parma University Hospital, Via Gramsci, Parma, Italy; Department of Medicine and Surgery, University of Parma, Parma, Italy; Department of Cardiology, Infermi Hospital, Rimini, Italy; School of Biomedical Engineering & Imaging Sciences, King’s College London, London, UK; De Gasperis’ Cardio Center, ASST Grande Ospedale Metropolitano Niguarda, Milan, Italy; Department of Cardiology, ASST Monza, P.O. Desio, Italy; Department of Radiology, ASST Papa Giovanni XXIII Hospital, 24127 Bergamo, Italy; Department of Perioperative Cardiology and Cardiovascular Imaging, Centro Cardiologico Monzino IRCCS, Via Carlo Parea, 4 , Milan 20138, Italy; Dipartimento Neuro-Cardiovascolare, Ospedale Ca’ Foncello Treviso, Treviso, Italy; Department of Diagnostic, Parma University Hospital, Via Gramsci, Parma, Italy; Cardiovascular Department, CMR Center, University Hospital Lausanne, CHUV, Lausanne, Switzerland; De Gasperis’ Cardio Center, ASST Grande Ospedale Metropolitano Niguarda, Milan, Italy; Department of Cardiac, Thoracic, Vascular Sciences and Public Health University of Padua Medical School, Padova, Italy; Department of Radiology, Careggi Hospital, Florence, Italy; Department of Diagnostic, Parma University Hospital, Via Gramsci, Parma, Italy; Division of Cardiology, Loyola University of Chicago, Chicago, IL, USA; Department of Cardiology, Edward Hines Jr. VA Hospital, Hines, IL, USA; Department of Cardiology, Citta` della Salute e della Scienza - Ospedale Molinette, Turin, Italy; Department of Cardiology, Hospital Universitari Vall d’Hebron, Barcelona, Spain; Vall d’Hebron Institut de Recerca (VHIR), Universitat Autònoma de Barcelona, Bellaterra, Spain; CIBER-CV, Instituto de Salud Carlos III (ISCIII), Madrid, Spain; Radiology Department, Vannini Hospital Rome, Rome, Italy; Division of Cardiovascular Imaging, Department of Radiology and Radiological Science, Medical University of South Carolina, Charleston, SC, USA; MariaCecilia Hospital, GVM Care & Research, Cotignola, RA, Italy; Department of Medicine and Surgery, University of Parma, Parma, Italy; Department of Radiology, University Hospital Leuven, Leuven, Belgium; Division of Cardiology, Loyola University of Chicago, Chicago, IL, USA; UOC Radiologia, Ospedale ‘F. Spaziani’, Frosinone, Italy; U.O.C. Risonanza Magnetica per Immagini, Fondazione G. Monasterio CNR-Regione Toscana Pisa, Pisa, Italy; Department of Radiology, Fondazione IRCCS Policlinico S. Matteo, Pavia, Italy; Division of Cardiovascular Imaging, Department of Radiology and Radiological Science, Medical University of South Carolina, Charleston, SC, USA; Department of Perioperative Cardiology and Cardiovascular Imaging, Centro Cardiologico Monzino IRCCS, Via Carlo Parea, 4 , Milan 20138, Italy; University Cardiology Unit, Interdisciplinary Department of Medicine, University of Bari Aldo Moro, Bari, Italy; Department of Radiology, ASST Papa Giovanni XXIII Hospital, 24127 Bergamo, Italy; Faculty of Medicine and Biology, University of Lausanne, UniL, Lausanne, Switzerland; Department of Perioperative Cardiology and Cardiovascular Imaging, Centro Cardiologico Monzino IRCCS, Via Carlo Parea, 4 , Milan 20138, Italy; Department of Biomedical, Surgical and Dental Sciences, University of Milan, Milan, Italy

**Keywords:** cardiac magnetic resonance, non-dilated left ventricular cardiomyopathy, dilated cardiomyopathy, sudden cardiac death

## Abstract

**Aims:**

Accurate risk stratification for patients with non-dilated left ventricular cardiomyopathy (NDLVC) remains challenging due to lack of dedicated clinical trials. This *post hoc* analysis aims to delineate the arrhythmic risk and assess the incremental value of cardiac magnetic resonance (CMR) imaging in the CarDiac magnEtic Resonance for prophylactic Implantable-cardioVerter defibrillAtor ThErapy (DERIVATE) study cohort meeting the NDLVC diagnostic criteria.

**Methods and results:**

Patients with NDLVC from the DERIVATE registry were identified in the absence of left ventricular (LV) dilatation and in the presence of non-ischaemic LV scarring (‘fibrotic NDLVC’) or isolated LV systolic dysfunction (LV ejection fraction < 50%) without fibrosis (‘hypokinetic NDLVC’). The primary endpoint was all-cause mortality. Major adverse arrhythmic cardiac events (MAACE) were the secondary endpoint and included sudden cardiac death (SCD) and aborted SCD. One hundred and ninety-seven NDLVC patients were identified from the cohort of the DERIVATE study (mean age: 59 ± 14 years; male: 135). Over a median follow-up of 2.7 years, 15 (8%) patients died and 8 (4%) experienced MAACE. Patients with ‘hypokinetic’ NDLVC had significantly lower rates of MAACE than non-ischaemic dilated cardiomyopathy (NIDCM) (*P* = 0.001), while patients with ‘fibrotic’ NDLVC had same rate of both primary (*P* = 0.48) and secondary endpoints (*P* = 0.616) compared with NIDCM patients. Multivariable analysis identified late gadolinium enhancement (LGE) with midwall distribution as an independent predictor of MAACE in NDLVC patients (hazard ratio 6.7, 95% confidence interval: 1.33–33.67; *P* = 0.021).

**Conclusion:**

NDLVC patients exhibit a heterogeneous risk profile for arrhythmic events. The presence of midwall LGE, similarly to NIDCM, is a significant predictor of MAACE, highlighting the importance of CMR imaging for risk stratification.

## Graphical Abstract

**Graphical Abstract:**
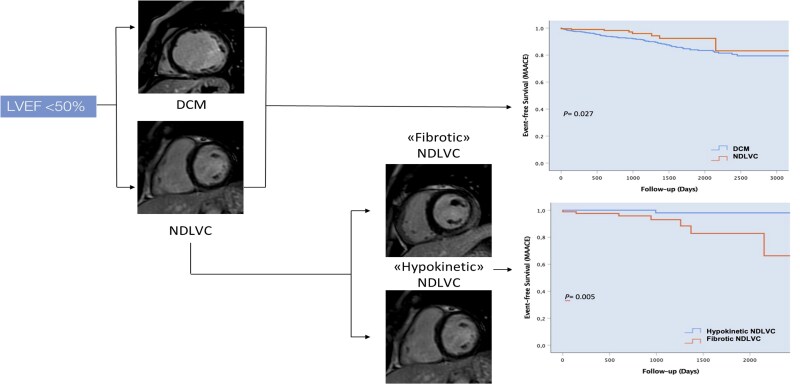
Risk of major adverse arrhythmic cardiac events in patients with dilated and non-dilated left ventricular cardiomyopathy. DCM, dilated cardiomyopathy; MAACE, major adverse arrhythmic cardiac event; NDLVC, non-dilated left ventricular cardiomyopathy.

**See the editorial comment for this article ‘NDLVC is just the entry door: advocating for an aetiology-oriented management’, by K. Galanti and C.A. Ahmed Chahal, https://doi.org/10.1093/ehjci/jeaf135.**

## Introduction

With an incidence markedly increasing with age, sudden cardiac death (SCD) accounts for ∼50% of all cardiovascular (CV) deaths and ∼10–20% of all deaths in Europe.^1,2^ Given the significant burden on mortality despite all the advances in clinical management, there is a need to establish novel risk stratification models to correctly identify patients who may benefit from prevention strategies and implantable cardioverter defibrillator (ICD) therapy. The guidelines-recommended approach is currently based on left ventricular ejection fraction (LVEF) estimation.^2^ However, the accuracy of this strategy seems to be reduced in the real-world scenario and insufficient to adequately stratify patients with both ischaemic dilated cardiomyopathy (IDCM) and non-IDCM (NIDCM).^3–5^ In 2016, Pinto *et al*.^6^ firstly suggested the introduction of a new category, the hypokinetic non-dilated cardiomyopathy, given the evidence that left ventricular (LV) dilatation can be very mild or even absent in some clinical scenarios, despite the evidence of myocardial disease at imaging assessment. The 2023 European Society of Cardiology (ESC) guidelines on cardiomyopathies^7^ later introduced a new clinical entity, the non-dilated left ventricular cardiomyopathy (NDLVC) phenotype; this is defined, in the absence of LV dilatation, either by (i) the presence of LV scarring with a non-ischaemic pattern or fatty replacement or by (ii) the presence of isolated LV systolic dysfunction (LVEF < 50%) with no overt underlying cause (i.e. abnormal loading conditions or coronary artery disease) and no myocardial scar.^6,7^ This heterogeneous group of patients has an increased risk of life-threatening arrhythmic events, and SCD risk prevention represents one of the cornerstones of their clinical management.^7,8^ Unfortunately, data on risk prediction and the usefulness of preventive strategies for NDLVC patients are lacking, and current recommendations for primary prevention of ICD implantation are based on the same LVEF criteria used for DCM patients.^7^ However, patients with NDLVC often have normal or only mildly impaired LV systolic function,^9^ and LVEF may be particularly inappropriate in identifying high-risk patients in this context. In addition, the two clinical entities included in the NDLVC definition may have different SCD burden, requiring a specific risk assessment approach. Recently, the CarDiac MagnEtic Resonance for Primary Prevention Implantable CardioVerter DebrillAtor ThErapy (DERIVATE) International Registry demonstrated that a cardiac magnetic resonance (CMR)–based risk score can provide incremental prognostic value over standard-of-care evaluation in a large cohort of NIDCM patients with LVEF < 50%.^10^ This *post hoc* analysis aims to characterize the arrhythmic risk and evaluate the additional prognostic value of CMR imaging in the DERIVATE study population fulfilling the NDLVC diagnostic criteria.

## Methods

### Study design and population

The design and main results of the DERIVATE study (http://www.clinicaltrials.gov: RCT#NCT03352648) have already been published.^10,11^ Briefly, this was an international, multicentric, prospective, observational registry enrolling patients referred for heart failure work-up from 21 sites across Europe and the USA. Patients with (i) age ≥ 18, (ii) diagnosis of chronic heart failure according to the ESC task force definition, and (iii) LVEF < 50% at initial transthoracic echocardiography (TTE) evaluation were enrolled. Exclusion criteria of the DERIVATE study were the presence of (i) IDCM, (ii) cardiomyopathies other than NIDCM, (iii) severe valvular heart disease, and (iv) congenital heart disease. According to the study protocol, patients underwent both TTE and CMR imaging within 3 months of enrolment, and clinical information was collected. For the purpose of our *post hoc* analysis, patients with NDLVC were identified in the whole cohort of the study using the diagnostic criteria proposed by ESC guidelines on cardiomyopathies.^7^ Specifically, NDLVC was defined in the absence of LV dilatation [i.e. LV end-diastolic volume indexed (LVEDVi) < 98 mL/m^2^ for men and <92 mL/m^2^ for women at CMR analysis^12^] and the presence of LV scarring with a non-ischaemic pattern or fatty replacement (‘fibrotic NDLVC’, irrespective of function), or isolated LV systolic dysfunction (LVEF < 50%) without scarring (‘hypokinetic NDLVC’). Dilated cardiomyopathy was defined in the presence of LV dilatation and systolic dysfunction.^13^ The primary endpoint of the study was all-cause mortality. Major adverse arrhythmic cardiac event (MAACE) was the secondary endpoint and included a combination of SCD and aborted SCD (either appropriate ICD shock, non-fatal cardiac arrest, or ventricular tachycardia (VT) lasting >30 s and/or causing haemodynamic instability).

### CMR protocol

Breath-hold cine steady-state free precession sequences were acquired for functional analysis and centrally examined by one observer using CMR4.2 software (Circle, Calgary, Canada). Late gadolinium enhancement (LGE) sequences were examined to identify the (i) presence of LGE, (ii) pattern and localization of LGE distribution as previously described,^14,15^ and (iii) number of LGE-positive segments. A standardized 17-segment model was used for the analysis. Non-ischaemic LGE included LGE with midwall, epicardial, and mixed distribution. We identified the presence of a ring-like pattern when at least three contiguous segments in the same short-axis slice were LGE positive.^16^

### Statistical analysis

Data analysis was performed using SPSS (IBM, USA), version 25.0. Continuous variables are expressed as mean ± standard deviation (SD) or median with interquartile range (IQR), as appropriate. Categorical variables are summarized as counts and percentages. Baseline characteristics were compared using the independent *t*-test or Mann–Whitney *U* test for continuous variables based on the normality of data distribution. For categorical variables, the *χ*^2^ test or Fisher’s exact test was used as appropriate. Univariable Cox proportional hazard models were used to identify predictors for the study endpoints, and predictors yielding a *P* < 0.05 were considered statistically significant and included in separate multivariable Cox proportional hazard models along with other clinically relevant variables. Survival analysis was conducted using the Kaplan–Meier method, and differences between groups were assessed using the log-rank test, considering statistically significant all *P* < 0.05.

## Results

### Study sample characteristics

Out of the entire DERIVATE study cohort, a subgroup of 197 patients (male: 68%, mean age 59 ± 14 years) with NDLVC was identified. Among them, 84 (43%) had non-ischaemic LGE (‘fibrotic’ NDLVC), and 113 (57%) had LV systolic dysfunction in the absence of LGE (‘hypokinetic’ NDLVC). Baseline patients’ characteristics are listed in *Tables 1–3*. A higher rate of beta-blockers and diuretic therapy prescription was observed in the DCM group (*P* < 0.001 for both), while NDLVC patients were more frequently on calcium blockers (*P* = 0.008). As expected, DCM patients had significantly larger volumes (*P* < 0.001) and higher LV mass index (*P* < 0.001) compared with NDLVC patients, with lower mean LVEF (32% vs. 41%, *P* < 0.001). The presence of non-ischaemic LGE was observed in 84 (43%) NDLVC patients and 545 (47%) DCM patients (*P* = 0.205); DCM patients had more LGE with midwall distribution [481 (42%) vs. 63 (32%), *P* = 0.009] and larger areas of non-ischaemic LGE (>3 segments) [350 (30%) vs. 35 (18%), *P* < 0.001] compared with NDLVC. No differences were observed in the prevalence of ring-like LGE among the two groups [133 (12%) vs. 17 (9%), *P* = 0.222], nor in the LGE localization within myocardial segments (*Table 2*). No significant differences were observed in terms of CMR parameters among the two subgroups of NDLVC (‘fibrotic’ and ‘hypokinetic’), except for a higher LV mass index in the ‘fibrotic’ group (70 ± 24 vs. 63 ± 15 g/m^2^, *P* = 0.031) (*Table 3*).

**Table 1:** Baseline clinical and echocardiographic characteristics in patients with DCM and NDLVC

Baseline characteristics	DCM (*n* = 1147)	NDLVC (*n* = 197)	*P*-value
Sex: male, *n* (%)	787 (68)	135 (68)	0.981
Age, year (mean ± SD)	56 ± 14	59 ± 14	**0.011**
Familiar history, *n* (%)	357 (31)	52 (26)	0.192
Hypertension, *n* (%)	450 (39)	91 (46)	0.06
Hyperlipidaemia, *n* (%)	371 (32)	60 (30)	0.593
Diabetes, *n* (%)	164 (14)	34 (17)	0.275
Beta-blockade, *n* (%)	973 (85)	146 (74)	**<0.001**
ACEi/ARB, *n* (%)	986 (86)	161 (82)	0.107
Diuretics, *n* (%)	755 (70)	99 (50)	**<0.001**
Calcium blockade, *n* (%)	45 (4)	17 (8)	**0.008**
Amiodarone, *n* (%)	157 (15)	21 (11)	0.144
Other anti-arrhythmic, *n* (%)	16 (1.7)	6 (3.1%)	0.170
TTE-LVEDVi (mL/m^2^), mean ± SD	107 ± 34	74 ± 36	**<0.001**
TTE-LVESVi (mL/m^2^), mean ± SD	73 ± 31	44 ± 18	**<0.001**
TTE-LVEF (%), mean ± SD	32 ± 11	40 ± 10	**<0.001**

All *p*-values <0.05 are highlighted in bold. ACEi, angiotensin-converting enzyme inhibitors; ARB, angiotensin receptor blockade; DCM, dilated cardiomyopathy; LVEDVi, left ventricle end-diastolic volume indexed; LVEF, left ventricle ejection fraction; LVESVi, left ventricle end-systolic indexed; NDLVC, non-dilated left ventricular cardiomyopathy; TTE, transthoracic echocardiography.

**Table 2:** Baseline CMR characteristics

Baseline CMR characteristics	DCM (*n* = 1147)	NDLVC (*n* = 197)	*P*-value
CMR-LVEDVi (mL/m^2^), mean ± SD	140 ± 38	83 ± 10	**<0.001**
CMR-LVESVi (mL/m^2^), mean ± SD	98 ± 38	49 ± 10	**<0.001**
CMR-LVEF (%), mean ± SD	32 ± 11	41 ± 8	**<0.001**
CMR-LVMi (g/m^2^), mean ± SD	84 ± 28	66 ± 19	**<0.001**
Non-ischaemic LGE (presence), *n* (%)	545 (47)	84 (43)	0.205
LGE midwall, *n* (%)	481 (42)	63 (32)	**0.009**
LGE subepicardial, *n* (%)	134 (12)	26 (13)	9.544
LGE > 3 segments, *n* (%)	350 (30)	35 (18)	**<0.001**
LGE septal, *n* (%)	443 (39)	64 (32)	0.101
LGE free wall, *n* (%)	299 (26)	44 (22)	0.267
LGE mixed (septal + free wall), *n* (%)	197 (17)	24 (12)	0.081
LGE ring-like, *n* (%)	133 (12)	17 (9)	0.222

All *p*-values <0.05 are highlighted in bold. CMR, cardiac magnetic resonance; DCM, dilated cardiomyopathy; LVEF, left ventricular ejection fraction; LVEDVi, left ventricular end-diastolic volume indexed; LVESVi, left ventricular end-systolic indexed; LGE, late gadolinium enhancement; LVMi, left ventricular mass indexed; TTE, transthoracic echocardiography; NDLVC, non-dilated left ventricular cardiomyopathy.

**Table 3:** Baseline clinical, TTE, and CMR characteristics in patients with hypokinetic and fibrotic NDLVC phenotype

Baseline characteristics	‘Hypokinetic’ NDLVC (*n* = 113)	‘Fibrotic’ NDLVC (*n* = 84)	*P*-value
Sex: male *n* (%)	75 (66)	60 (71)	0.450
Familiar history, *n* (%)	31 (27)	21 (26)	0.815
Hypertension, *n* (%)	53 (47)	38 (46)	0.831
Hyperlipidaemia, *n* (%)	37 (32)	23 (28)	0.450
Diabetes, *n* (%)	19 (17)	15 (18)	0.818
Age, year (mean ± SD)	58 ± 15	59 ± 14	0.628
TTE-LVEDVi (mL/m^2^), mean ± SD	72 ± 24	76 ± 25	0.329
TTE-LVESVi (mL/m^2^), mean ± SD	43 ± 18	45 ± 18	0.387
TTE-LVEF (%), mean ± SD	40 ± 10	40 ± 11	0.773
CMR-LVEDVi (mL/m^2^), mean ± SD	82 ± 11	85 ± 9	0.7
CMR-LVESVi (mL/m^2^), mean ± SD	49 ± 10	49 ± 9	0.916
CMR-LVEF (%), mean ± SD	40 ± 7	42 ± 9	0.168
CMR-LVMi (g/m^2^), mean ± SD	63 ± 15	70 ± 24	**0**.**031**
CMR-RVEDVi (mL/m^2^), mean ± SD	62 ± 17	65 ± 19	0.547
CMR-RVESVi (mL/m^2^), mean ± SD	30 ± 13	31 ± 13	0.554
CMR-RVEF (%), mean ± SD	53 ± 10	52 ± 11	0.407

All *p*-values <0.05 are highlighted in bold. CMR, cardiac magnetic resonance; LVEDVi, left ventricle end-diastolic volume indexed; LVEF, left ventricle ejection fraction; LVESVi, left ventricle end-systolic indexed; RVEDVi, right ventricle end-diastolic volume indexed; RVEF, right ventricle ejection fraction; RVESVi, right ventricle end-systolic indexed; NDLVC, non-dilated left ventricular cardiomyopathy; TTE, transthoracic echocardiography.

### Risk of events in patients with DCM and NDLVC

The rates of events are listed in *Table 4*. Over a median follow-up of 2.7 years (IQR: 1.6–4.4 years), 15 NDLVC (8%) and 73 DCM (6%) patients died (*P* = 0.532). Patients with DCM experienced more MAACE compared with patients with NDLVC [115 (10%) vs. 8 (4%), *P* = 0.007]. In detail, patients with DCM had more ICD implantations, more appropriate discharge [420 (37%) vs. 25 (13%) and 79 (8%) vs. 1 (0.5%), both *P* < 0.001] and sustained VT [92 (9%) vs. 6 (3%), respectively, *P* = 0.005), while no significant difference was observed in terms of rate of non-fatal cardiac arrest (<1% for both, *P* = 0.639) and SCD [13 (1%) vs. 1 (0.5%), *P* = 0.423]. When looking at the ‘fibrotic’ NDLVC subgroup, there was no difference in the rate of both primary and secondary endpoints compared with patients with DCM (*Table 4*; *P* = 0.48 and *P* = 0.616, respectively). Patients with ‘hypokinetic’ NDLVC had instead similar rates of death for all causes compared with DCM patients [8 (7%) vs. 73 (6%), *P* = 0.767) but significantly lower rates of MAACE [1 (0.9%) vs. 115 (10%), *P* = 0.001], mainly driven by a lower rate of ICD appropriate discharge and sustained VT in this population (*Table 4*; *P* = 0.002 and *P* < 0.001, respectively).

**Table 4:** Rate of cardiac events in DCM vs. NDLVC

Cardiac events	DCM (*n* = 1147)	NDLVC (*n* = 197)	Fibrotic NDLVC (*n* = 84)	Hypokinetic NDLVC (*n* = 113)	DCM vs. NDLVC	DCM vs. fibrotic NDLVC	DCM vs. hypokinetic NDLVC	Fibrotic NDLVC vs. hypokinetic NDLVC
	(*n*, %)	(*n*, %)	(*n*,%)	(*n*,%)	*P*-value	*P*-value	*P*-value	*P*-value
All causes of death	73 (6)	15 (8)	7 (8)	8 (7)	0.532	0.480	0.767	0.743
MAACE	115 (10)	8 (4)	7 (8)	1 (0.9)	**0.007**	0.616	**0.001**	**0.009**
ICD implantation	420 (37)	25 (13)	13 (15)	12 (10)	**< 0.001**	**<0.001**	**<0.001**	0.311
Hospitalization for HF	235 (20)	19 (10)	8 (9)	11 (10)	**0.001**	**0.046**	**0.019**	0.960
ICD appropriate discharge	79 (8)	1 (0.5)	1 (1)	0 (0)	**< 0.001**	**0.028**	**0.002**	0.245
NSVT	209 (20)	17 (9)	9 (11)	8 (7)	**<0.001**	0.104	**0.003**	0.369
SVT	92 (9)	6 (3)	6 (7)	0 (0)	**0.005**	0.581	**<0.001**	**0.004**
Non-fatal cardiac arrest	8 (0.8)	1 (0.5)	0 (0)	1 (0.9)	0.639	0.401	0.948	0.385
Cardiac death	46 (4)	6 (3)	3 (4)	3 (3)	0.515	0.841	0.476	0.711
Cardiac death for HF	25 (2)	5 (2)	2 (2)	0 (0)	0.754	0.904	0.255	0.245
SCD	13 (1)	1 (0.5)	1 (1)	3 (3)	0.423	0.963	0.745	0.904

All *p*-values <0.05 are highlighted in bold. DCM, dilated cardiomyopathy; HF, heart failure; ICD, implantable cardioverter defibrillator; NDLVC, non-dilated left ventricular cardiomyopathy; MAACE, major adverse arrhythmic cardiac events; NSVT, non-sustained ventricular tachycardia; SCD, sudden cardiac death; SVT, sustained ventricular tachycardia

The primary endpoint reached the same rate among the two subgroups of NDLVC (*Table 4*; *P* = 0.743). However, MAACE and sustained VT were more prevalent in patients with ‘fibrotic’ NDLVC (*P* = 0.009 and *P* = 0.004, respectively).

### Predictors of all-cause mortality and MAACE in NDLVC patients

The predictors of primary and secondary endpoint are listed in *Table 5*. At univariable analysis, diabetes mellitus and several tissue characteristics measured by CMR, including the presence of LGE in the LV free wall, the extent of LGE (total number of segments with midwall LGE and presence of non-ischaemic LGE in more than three myocardial segments), a ring-like LGE pattern, and the presence of LGE in both septum and free wall, were associated with the primary endpoint. In a multivariable analysis model, all variables, except isolated LV free-wall LGE, remained independent predictors after controlling for clinically meaningful confounders such as age and LVEF. A trend was also observed for the number of segments with midwall LGE, although it did not reach statistical significance. Amiodarone prescription, presence of non-ischaemic LGE, and LGE with midwall distribution were instead significantly associated with MAACE in both univariable and multivariable analyses.

**Table 5:** Univariable and multivariable predictors of primary and secondary endpoints in NDLVC patients

	Univariable predictors				Multivariable predictors			
	All cause of death		MAACE		All cause of death		MAACE	
	HR (95% CI)	*P*-value	HR (95% CI)	*P*-value	HR (95% CI)	*P*-value	HR (95% CI)	*P*-value
Demographic characteristics								
Age (years)	1.015 (0.987–1.054)	0.427	1.260 (0.301–5.273)	0.752				
Male	1.046 (0.357–3.064)	0.934	0.688 (0.138–3.432)	0.648				
Cardiovascular risk factor								
Family history	0.9 (0.29–2.86)	0.872	0.9 (0.29–2.86)	0.872				
Smoking history	0.5 (0.14–1.89)	0.328	0.6 (0.13–3.43)	0.648				
Hypertension	0.7 (0.25–2.04)	0.540	0.6 (0.15–2.70)	0.546				
Hyperlipidaemia	1.7 (0.63–5.03)	0.276	0.8 (0.16–4.18)	0.832				
Diabetes	3.7 (1.25–11.29)	0.018	1.03 (0.12–8.62)	0.976	**4.2 (1.32–13.46)**	**0**.**015**		
NYHA class (III–IV)	1.8 (0.52–6.79)	0.330	2.6 (0.52–13.85)	0.238				
Pharmacological therapy								
Beta-blockers	1.07 (0.34–3.40)	0.902	2.5 (0.31–21.26)	0.375				
Amiodarone	1.3 (0.301–6.03)	0.696	10.7 (2.39–47.99)	0.002			**9.9 (2.09–47.61)**	**0**.**004**
Antithrombotic agents	0.9 (0.35–2.80)	0.986	1.4 (0.37–6.03)	0.572				
Anticoagulant therapy	0.9 (0.27–3.54)	0.994	1.2 (0.25–6.24)	0.777				
TTE								
LVEDVi (mL/m^2^)	0.9 (0.95–1)	0.150	1 (0.9–1.04)	0.645				
LVEF (%)	1 (0.96–1.05)	0.750	1 (0.9–1.07)	0.838				
TAPSE (mm)	0.9 (0.79–1.03)	0.153	1.07 (0.88–1.31)	0.448				
CMR functional evaluation								
LVEDVi (mL/m^2^) (per 1 mL/m^2^)	0.9 (0.93–1.02)	0.281	1.02 (0.94–1.107)	0.602				
LVESVi (mL/m^2^) (per 1 mL/m^2^)	0.9 (0.92–1.01)	0.206	1.01 (0.95–1.08)	0.636				
LVMi (g/m^2^) (per 1 g/m^2^)	0.9 (0.96–1.01)	0.468	0.9 (0.92–1.06)	0.833				
LVEF (per point %)	1.02 (0.97–1.08)	0.348	0.9 (0.94–1.02)	0.486				
RVEDVi (mL/m^2^) (per 1 mL/m^2^)	1.01 (0.98–1.04)	0.398	0.95 (0.90–1.01)	0.118				
RVEF (per point %)	0.9 (0.95–1.03)	0.760	1.05 (0.95–1.15)	0.290				
CMR LGE evaluation								
Presence of non-ischaemic LGE	1.2 (0.45–3.47)	0.661	10.8 (1.33–88.97)	0.026			10.9 (1.33–90.13)	0.026
LGE septum	1.8 (0.67–5.18)	0.226	3.5 (0.85–14.98)	0.082				
LGE free wall	2.8 (1–8.01)	0.049	2.8 (0.67–12.06)	0.155	2.5 (0.81–8.28)	0.109		
LGE combined (septal + free wall)	6 (2.1–17.2)	0.001	7.2 (0.45–116.28)	0.161	**6.7 (2.07–22.15)**	**0.002**		
Presence of midwall LGE	1.4 (0.50–4.04)	0.503	6.7 (1.34–33.77)	0.020			**6.7 (1.33–33.67)**	**0**.**021**
Presence of epicardial LGE	0.5 (0.07–4.15)	0.555	1.1 (0.13–9.4)	0.910				
No. of segments with mixed LGE (per 1 segment)	1.07 (0.9–1.24)	0.360	1.05 (0.84–1.29)	0.656				
No. of segments with midwall LGE (per 1 segment)	1.2 (1.01–1.55)	0.035	0.7 (0.25–2.28)	0.632	1.2 (0.89–1.59)	0.064		
No. of segments with epicardial LGE (per 1 segment)	0.9(0.71–1.34)	0.914	1.1 (0.92–1.53)	0.171				
Presence of non-ischaemic LGE > 3 myocardial segments	3.4 (1.23–9.78)	0.019	1.7 (0.34–8.54)	0.510	**3.4 (1.14–10.65)**	**0**.**028**		
LGE ring-like pattern	5.4 (1.85–16.07)	0.002	1.5 (0.19–12.81)	0.671	**5.7 (1.69–19.19)**	**0**.**005**		

All *p*-values <0.05 are highlighted in bold. CMR, cardiac magnetic resonance; LGE, late gadolinium enhancement; LVEDVi, left ventricle end-diastolic volume indexed; LVEF, left ventricle ejection fraction; LVESVi, left ventricle end-systolic indexed; LVMi, left ventricle mass indexed; RVEDVi, right ventricle end-diastolic volume indexed; RVEF, right ventricle ejection fraction; RVESVi, right ventricle end-systolic indexed; NDLVC, non-dilated left ventricle cardiomyopathy; TAPSE, tricuspid annular plane systolic excursion; TTE, transthoracic echocardiography

### Survival analysis

**Figure 1:**
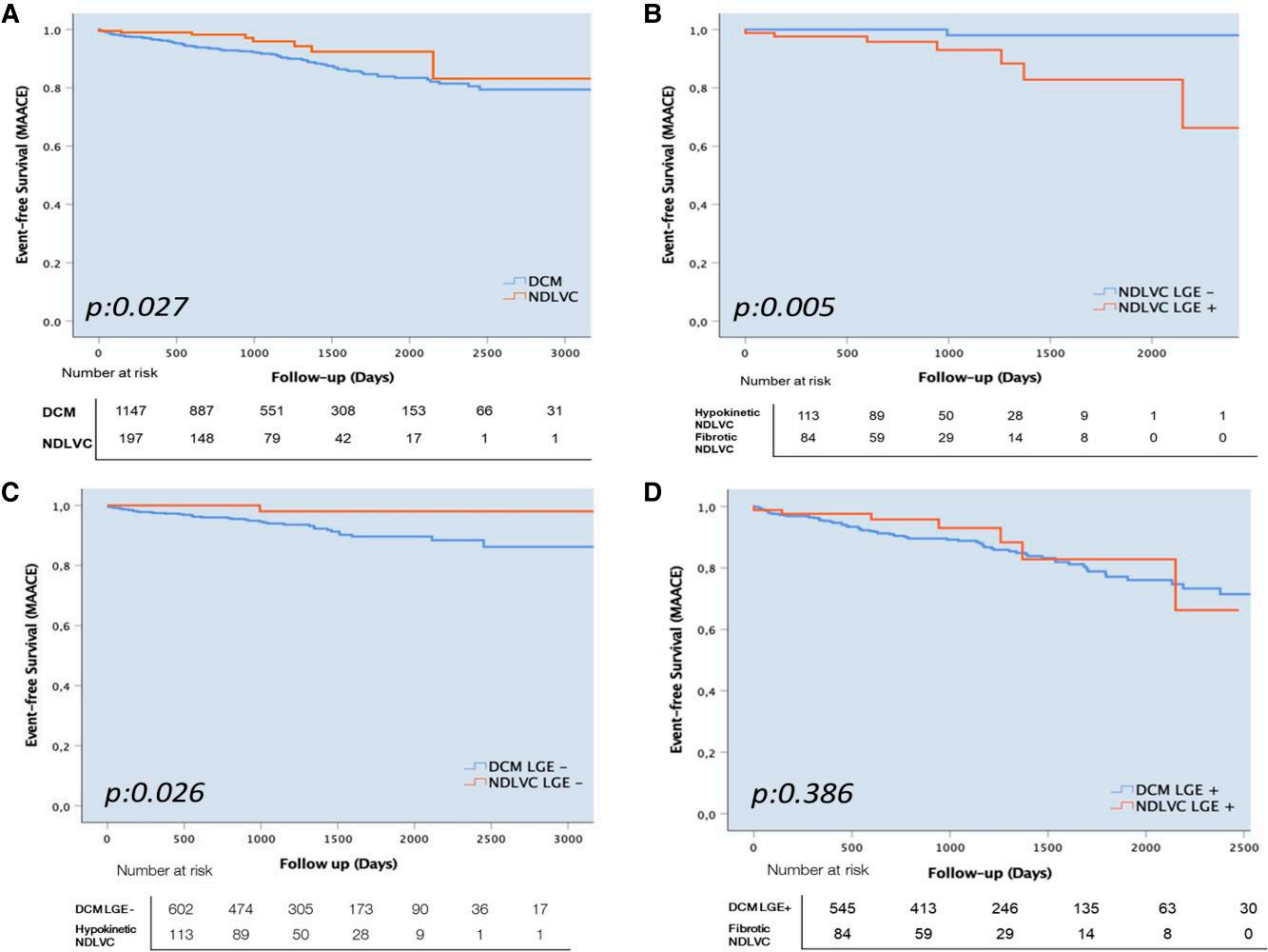
Kaplan–Meier curves for secondary endpoints in DCM and NDLVC patients (*A*) and in NDLVC with and without LGE (*B*). Kaplan–Meier curves for secondary endpoints in DCM and NDLVC LGE-negative (*C*) and LGE-positive (*D*) patients. DCM, dilated cardiomyopathy; LGE, late gadolinium enhancement; MAACE, major adverse arrhythmic cardiac event; NDLVC, non-dilated left ventricular cardiomyopathy.

The Kaplan–Meier curves for MAACE demonstrated that patients with NDLVC had a lower likelihood of reaching the study endpoint than DCM patients [hazard ratio (HR): 0.45; 95% confidence interval (CI): 0.22–0.93; log-rank test, *P* = 0.027; *Figure 1A*]. However, there was a substantial difference between the two NDLVC subgroups (*Figure 1B*). Indeed, the risk of MAACE was significantly higher in DCM compared with ‘hypokinetic’ NDLVC patients (HR: 10.7, 95% CI: 1.49–76.88; log-rank test, *P* = 0.003), even when considering only DCM patients without LGE (HR: 6.9, 95% CI: 0.95–50.64; *P* = 0.026; *Figure 1C*). Importantly, there was no difference between ‘fibrotic’ NDLVC and LGE-positive DCM (HR 0.71, 95% CI: 0.32–1.54; *P* = 0.386; *Figure 1D*). Patients with ‘fibrotic’ NDLVC also had a 10-fold higher risk of MAACE compared with ‘hypokinetic’ NDLVC (HR 10.89; 95% CI: 1.33–88.97; *P* = 0.005; *Figure 1B*).

## Discussion

The main results of this *post hoc* analysis can be summarized as follows: (i) the prevalence of MAACE is significantly different among DCM and NDLVC patients, occurring in only 4% of all NDLVC patients compared with 10% in the DCM group; (ii) MAACE risk among the NDLVC population is not homogenous, with more arrhythmic events described in the ‘fibrotic’ group; (iii) in NDLVC patients, the presence of midwall LGE is an independent predictor of MAACE (*Graphical Abstract*) while the presence of combined (septal and free-wall) LGE, presence of LGE in >3 segments, and a ring-like LGE pattern are independent predictors of all-cause death.

The introduction of the NDLVC phenotype aimed to address limitations in the traditional DCM definition, identifying patients with early/intermediate phenotypes who do not meet classical diagnostic criteria but still exhibit myocardial disease.^7^ This novel classification includes patients with non-ischaemic LV scarring or fatty replacement (‘fibrotic’ NDLVC) and those with isolated global LV hypokinesia without myocardial scarring (‘hypokinetic’ NDLVC). Although the same LVEF-based criteria are recommended to guide preventive ICD implantation in both DCM and NDLVC, these patients have different risk profiles. We found that the risk of MAACE was significantly lower in NDLVC patients, due to lower rate of sustained VT and appropriate ICD discharge (although the rate of ICD implantation was also lower in this population). Moreover, the arrhythmic risk may vary substantially within the heterogeneous NDLVC population. Patients with ‘fibrotic’ NDLVC had increased likelihood of experiencing MAACE than ‘hypokinetic’ NDLVC, with a risk profile very similar to DCM patients. In our cohort, non-ischaemic LGE, particularly LGE with midwall distribution, was significantly associated with major arrhythmic events at follow-up. The key role of LGE in risk stratification is not surprising and already established in previous studies for DCM patients,^17–19^ also in patients with only mild/moderate systolic dysfunction.^20^ The DERIVATE study itself proved how implementing LGE information in a composite risk score increased the accuracy of prognostic assessment in DCM patients beyond standard of care evaluation.^10^ Patients with NDLVC may particularly benefit from this CMR-based risk assessment approach. The results of the DANISH (Danish Study to Assess the Efficacy of ICDs in Patients with Non-ischemic Systolic Heart Failure on Mortality) trial failed to demonstrate a survival benefit of primary ICD implantation in DCM patients, suggesting the need of more accurate identification of patients that are at increased risk of arrhythmic events, but at lower risk to die for other, non-arrhythmic, causes.^4^ Patients with NDLVC, who often exhibit only mild systolic dysfunction,^9^ are less likely to experience limiting heart failure symptoms. This makes them an ideal population to potentially benefit from ICD protection for a more extended period of time.

Despite the presence of LGE was an independent predictor of MAACE, we found no association between LGE location or extension and major arrhythmic events. A non-linear relationship between LGE and SCD risk has already been demonstrated in DCM patients and, more recently, in NDLVC.^8,20,21^ In our analysis, LGE with a ring-like pattern and the presence of LGE in both septum and LV free wall were instead associated with all-cause mortality. An association between combined septal and free-wall LGE and death for all causes was already found by Halliday *et al*.^15^ in a cohort of 874 DCM patients. However, those authors found that this combined LGE distribution was most associated with SCD, while septal LGE was associated with all-cause mortality.^15^ The association between combined septal and free-wall LGE and major arrhythmic events was later confirmed in a larger cohort (*n* = 1165) of DCM patients. This study also showed higher arrhythmic risk in patients with epicardial or transmural LGE. However, data about the exact role of LGE location are conflicting; a recently published study^8^ proved that despite a higher prevalence of free-wall LGE observed in NDLVC, only septal LGE location was an independent predictor of major arrhythmic events and SCD. The clinical implications subtended by these results are of utmost importance. If CMR already has a pivotal role in the 2023 ESC guidelines,^7^ LGE assessment can be particularly crucial for the heterogeneous group of NDLVC patients. Identifying patients with LGE (‘fibrotic’), indicating higher risk of arrhythmic events, may allow to refine tailored preventive strategies, beyond LVEF assessment.

## Limitations

This is a *post hoc*, secondary analysis of the DERIVATE study. As such, the study was not adequately powered and further studies with larger, pre-specified sample sizes are needed to confirm these findings. In addition, the retrospective design of the study restricts its ability to establish causality. Despite ESC guidelines defining the presence of LV dilatation based on echocardiographic parameters, specific CMR cut-offs have been used to identify NDLVC for the purpose of our analyses. We had no data about other imaging findings (i.e. evidence of inflammation at nuclear imaging) or about the genotype of patients enrolled in the DERIVATE study. However, recently published data on NDLVC patients, including a comprehensive genetic characterization, demonstrated that the presence of LGE was associated with increased risk of event independently from the genetic status.^8^ Finally, the relatively small number of events in the NDLVC subgroups may affect the generalizability of the findings. Further prospective studies with larger cohorts are needed to validate these results and refine risk stratification models for NDLVC patients.

## Conclusions

NDLVC patients have a lower risk of arrhythmic events compared with DCM patients, with variability between NDLVC subgroups. ‘Fibrotic’ NDLVC patients have similar risks to DCM patients, while ‘hypokinetic’ NDLVC patients have a significantly lower risk. The presence of LGE, particularly with midwall distribution, is an independent predictor of arrhythmic events, suggesting that CMR imaging should be used for better risk stratification in NDLVC patients. Further large-scale studies are needed to confirm these findings and improve risk models for this subset of patients.

## Data Availability

The data underlying this article will be shared on reasonable request to the corresponding author.
